# Analysis of ORF5 sequences of Porcine Reproductive and Respiratory Syndrome virus (PRRSV) circulating within swine farms in Costa Rica

**DOI:** 10.1186/s12917-021-02925-7

**Published:** 2021-06-12

**Authors:** Mónica Guzmán, Ronald Meléndez, Carlos Jiménez, Marta Piche, Emily Jiménez, Bernal León, Juan M. Cordero, Lisbeth Ramirez-Carvajal, Alberto Uribe, Arie Van Nes, Arjan Stegeman, Juan José Romero

**Affiliations:** 1 Department of Veterinary Diagnostics (DDV), Veterinary Services National Laboratories (LANASEVE), Animal Health National Service (SENASA), Ministry of Livestock and Agriculture (MAG), Heredia, Costa Rica; 2 Department of Population Health Sciences, University of Utrecht, Utrecht, The Netherlands; 3 Consultoría Regional de Investigación en Producción Animal Sostenible (CRIPAS), School of Veterinary Medicine (EMV), Universidad Nacional (UNA), Heredia, Costa Rica; 4 Department of Virology, School of Veterinary Medicine (EMV), Universidad Nacional (UNA), Heredia, Costa Rica; 5 Faryvet Company, Heredia, Costa Rica; 6 Boehringer Ingelheim, 16300 Xochimilco, Mexico

**Keywords:** Porcine Reproductive and Respiratory Syndrome (PRRS), phylogenetic tree, Open reading frame 5 (ORF5), Glycoprotein 5(GP5), Costa Rica, Porcine Respiratory Disease Complex (PRDC)

## Abstract

**Background:**

Worldwide, Porcine Reproductive and Respiratory Syndrome (PRRS) is among the diseases that cause the highest economic impact in modern pig production. PRRS was first detected in Costa Rica in 1996 and has since then severely affected the local swine industry. Studies of the molecular characterization of circulating strains, correlation with clinical records, and associations with pathogens associated with Porcine Respiratory Disease Complex (PRDC) have not been done in Costa Rica.

**Results:**

Sequencing and phylogenetic analysis of ORF5 proved that PRRSV-2 was the only species detected in all locations analyzed. These sequences were grouped into three clusters. When comparing samples from San Jose, Alejuela, and Puntarenas to historical isolates of the previously described lineages (1 to 9), it has been shown that these were closely related to each other and belonged to Lineage 5, along with the samples from Heredia. Intriguingly, samples from Cartago clustered in a separate clade, phylogenetically related to Lineage 1. Epitope analysis conducted on the GP5 sequence of field isolates from Costa Rica revealed seven peptides with at least 80% amino acid sequence identity with previously described and experimentally validated immunogenic regions. Previously described epitopes A, B, and C, were detected in the Santa Barbara-Heredia isolate.

**Conclusions:**

Our data suggest that the virus has three distinct origins or introductions to the country. Future studies will elucidate how recently introduced vaccines will shape the evolutionary change of circulating field strains.

**Supplementary Information:**

The online version contains supplementary material available at 10.1186/s12917-021-02925-7.

## Background

Porcine reproductive and respiratory syndrome (PRRS) has been reported among the diseases with the highest economic impact in modern pig production [1]. The disease has a worldwide distribution and is endemic in most pig-producing countries. It is characterized by reproductive failure in late gestation, as well as by loss of weight gain, and poor performance in finishing pigs, and respiratory disorders [2–5]. PRRS viruses (PRRSV) can persist in affected farms for years, exacerbating the associated chronic animal health effects and economic losses due to this disease. For instance, in the USA, PRRS has caused productivity losses in breeding and growing-pig herds estimated at a $664 million loss annually [6]. Different studies have described losses in the breeding herd or growing-pig herd ranging from 12% to 45% of the total economic cost of PRRSV [7]. The emergence of highly pathogenic PRRSV strains from China has exacerbated global food insecurity risk [8].

PRRSV are enveloped, single-stranded, positive-sense RNA stranded, members of the *Arteriviridae* family, genus *Porartevirus* [9]. According to the International Committee on Taxonomy of Viruses (ICTV), two different species of PRRSV have been reported, PRRSV-1 (formerly known as European, EU) and PRRSV-2 (formerly known as North American, NA). General clinical signs, disease phenotype, genomic organization, and temporal emergence are all similar between the two species. However, both species are antigenically and genetically diverse [10, 11]. This high degree of genetic variability evidenced that both species are continuously evolving to adapt to existing immunity where they can re-emerge as new variants with the potential to cause outbreaks [12].

Retrotranscription followed by PCR has been used to differentiate formerly known EU and NA genotypes [10], and molecular epidemiology studies have been carried out by conducting the phylogenetic analysis of specific structural genes. These studies proved that both PRRSV species contain a genome of approximately 15 kb encoding 10 open reading frames (ORF): ORF1a, ORF1b, ORF2a, ORF2b, and ORFs 3-7, which include ORF5 and ORF5a [13] . The major structural proteins GP5, protein membrane (M), and protein N are encoded by ORF5 to ORF7. GP5 is a transmembrane glycosylated protein [14] and is the most heterogeneous, with 88% to 99% amino acid identity among strains of the same species, and 52% to 60% nucleotide identity between PRRSV species [8].

Phylogenetic analyses are based primarily on ORF5 which is the most examined gene and one of the most variable regions of the genome [15–17]. However, taxonomic emphasis on ORF5 could hinder significant genetic variation in other regions of the PRRSV genome [18]. Although the origin of PRRSV is unknown, the widening differences between PRRSV-1 and PRRSV-2 suggest that their ancestor evolved independently in different ecological or geographical means over an extended period of time, possibly in a non-pig reservoir [18]. PRRSV have been further grouped into 9 lineages (PRRSV-2) and 4 subtypes (PRRSV-1) based upon ORF5 phylogenetic relationships [19].

Several modified live virus (MLV), inactivated, DNA, subunit, and virus-vectored vaccines have been launched against both PRRSV-1 and PRRSV-2. These vaccines are registered in various countries depending on circulating viral genotypes (reviewed in [8]), but current vaccines against PRRSV have several drawbacks. In the case of the first commercially available vaccine, Ingelvac PRRS® MLV, the prevalence of PRRSV infection in swine herds is still high despite being widely used [20]. The vaccines are most effective if they are homologous to the field virus [21].

During PRRSV infections, the host develops a prolonged viremia followed by persistent infection in lymphoid tissues, indicating that the host immune system does not effectively clear the infection [22]. A meagre induction of innate immunity is detected, including a slow appearance of virus-specific gamma interferon (IFN-γ) [23–25]. A hallmark of the swine antibody response against PRRSV is a weak and delayed development of neutralizing antibodies (NAbs) (not sooner than 3 weeks after infection) in contrast with the abundant non-neutralizing antibodies (NNA) detected in early infection [26].

The role of NAbs in protective immunity and its importance for PRRSV vaccine development is demonstrated by the fact that passive transfer of NAbs before challenge with the homologous virulent PRRSV strain is sufficient to achieve complete protection against infection [27, 28]. Results from different laboratories have stressed the importance of finding effective epitopes to understand PRRSV pathogenesis and to develop a protective immune response against it by eliciting NAs that provide sufficient protection against infection [26, 29].

Despite the economic importance of PRRS and its high prevalence in Costa Rica, molecular characterization of species circulating in the country has not been carried out. This study aimed to improve understanding of the geographic and temporal distribution of PRRSV circulating within breeding and pig production farms and identify potential immunogenic targets. For the first time, we obtained sequences of PRRSV-2 ORF5 from Costa Rica and conducted phylogenetic and epitope analyses. We anticipate that the molecular characterization of PRRS species circulating within swine farms in Costa Rica will guide decision-making processes regarding the implementation of vaccination programs based on viral genetic information.

## Results

### Study Farms

In Table 1 we describe the information of the samples included in this study. We recorded the type, size, location of the farm, confirmed diagnostics of pathogens detected simultaneously as PRRSV detection in 2019. We included historical records provided by the field veterinarian related to the first description of PRDC in each farm and lab results of pathogens detected at the same time as PRDC. All farms involved were breeding farms and there were mostly large and medium farms.

**Table 1:** Respiratory pathogens present during sampling for PRRSV detection and historical clinical and laboratory records

Location	Production system	Farm Size	Number of sows	Associated to isolates from 2019	Associated to earlier PRDC reports in farms	
				Pathogens detected	First description of PRDC^a^	Pathogens associated confimed by lab
Cartago	Breeders	Large	3500	*Haemophilus* spp-Porcine circovirus 2 (PCV2)	1993	PRRSV and PCV2
Puntarenas	Breeders	Medium	225	*Glaesserella parasuis and Actinobacillus pleuropneumoniae (*APP*)* Influenza	1996, 2018	PRRSV and PCV2
Puriscal	Breeders	Medium	200	*Mycoplasma spp* -APP	2015	PRRSV, APP, *Mycoplasma spp*
San Ramon	Breeders	Medium	190	*Glaesserella parasuis, Mycoplasma spp -* PCV2	2015	PRRSV and PCV2
San Ramon	Breeders	Medium	240	*Mycoplasma spp* -APP	2015	PRRSV and PCV2
Santa Barbara	Breeders	Medium	220	*Mycoplasma spp* -APP- PCV2	2002, 2015	(2002: PRRSV and *Glaesserella parasuis*) (2015: PRRSV and PCV2)

^a^ Post-weaning Respiratory Disease Complex (PRDC), first description and outbreaks

For the oldest farm (Coris, Cartago), clinical records dating back to 1993 were available, including the history of semen and breeders imports, major pathogens detected, productive and reproductive parameters.

### Virus geographical and temporal occurrence related to economically relevant pathogens

PRRSV is an important agent in the presentation of respiratory problems in pigs. However, the clinical presentation may be complicated by other viral agents such as Porcine Circovirus type 2, Influenza virus or bacteria such as *Haemophilus spp*, *Glaesserella parasuis*, *Actinobacillus pleuropneumoniae* (APP), and *Mycoplasma spp* [30–32]. Five farms reported cases of bacterial and viral respiratory pathogens in addition to the detection of PRRSV (Table 1). Information from historical clinical records showed the presence of Porcine Respiratory Disease Complex (PRDC) and the laboratory detection of PRRSV in all farms as early as 1993 (Table 1).

### Epitope analysis of field strains from Costa Rica

We found seven different epitopes represented in at least one of the isolates from Costa Rica with amino acid sequence identity ranging from 80% to 100% (Table 2) when compared to validated and previously published epitopes [33]. These matching peptides were distributed among numerous locations of GP5. The Gp5-KK (KGRLYRWRSPVIVEK) was found in all isolates. The Gp5-SL (SHLQLIYNL) motif was the second most frequently detected (5 out of 7) isolate, followed by Epitope C which was found in 4 out of 7 isolates. Interestingly, epitope A, B, and C were all found in an isolate from Santa Barbara-Heredia (Table 2).

**Table 2:** Epitopes identified in GP5 sequences from Costa Rica PRRSV-2 isolates Phylogenetic analysis of ORF5

Isolate from Costa Rica	Start	End	Match	Length of Match	Identity (%)	Epitope ID and Reference fron literature
RA5_Puriscal-SanJose-CR_2019	29	37	SQLQLIYNL	9	88.9	Gp5-SL,Ostrowski et al. (2002), PRRSV2
	141	155	KGKLYRWRSPVIIEK	15	86.7	Gp5-KK, Vashisht et al. (2008), PRRSV2, aa149–163
	179	192	TPITKVSAEQWGRP	14	85.7	Gp5-TP, de Lima et al. (2006), PRRSV 2. aa 187–200
	44	53	GTEWLAGKFD	10	80.0	Epitope C Guo et al. 2019, 52-61aa
C4_Corredores-Puntarenas-CR_2019	37	45	SQLQLIYNL	9	88.9	Gp5-SL,Ostrowski et al. (2002), PRRSV2
	117	131	LAAFICFIIRAAKNC	15	80.0	Gp5-LC, Vashisht et al. (2008), PRRSV2, aa 117–131
	149	163	KGRLYRWRSPVIIEK	15	93.3	Gp5-KK, Vashisht et al. (2008), PRRSV2, aa149–163
	187	200	TPITKVSAEQWGRP	14	85.7	Gp5-TP, de Lima et al. (2006), PRRSV 2. aa 187–200
	52	61	GTDWLAGKFD	10	90.0	Epitope C Guo et al. 2019, 52-61aa
D8_SanRamon-Alajuela-CR_2019	37	45	SQLQLIYNL	9	88.9	Gp5-SL,Ostrowski et al. (2002), PRRSV2
	149	163	KGKLYRWRSPVIIEK	15	86.7	Gp5-KK, Vashisht et al. (2008), PRRSV2, aa149–163
	187	200	TPITKVSAEQWGRP	14	85.7	Gp5-TP, de Lima et al. (2006), PRRSV 2. aa 187–200
	52	61	GTDWLAGKFD	10	90.0	Epitope C Guo et al. 2019, 52-61aa
R2_SantaBarbara-Heredia-CR_2019	37	45	SHLQLIYNL	9	100.0	Gp5-SL,Ostrowski et al. (2002), PRRSV2
	117	131	LAALTCFTIRFAKNC	15	80.0	Gp5-LC, Vashisht et al. (2008), PRRSV2, aa 117–131
	149	163	KGRLYRWRSPVIIEK	15	93.3	Gp5-KK, Vashisht et al. (2008), PRRSV2, aa149–163
	187	200	TPVTRVSAEQWGRP	14	92.9	Gp5-TP, de Lima et al. (2006), PRRSV 2. aa 187–200
	27	30	VLVN	4	100.0	Epitope A, Guo et al. 2019, 27-30 aa
	52	61	GTDWLAGKFD	10	90.0	Epitope C Guo et al. 2019, 52-61aa
	37	45	SHLQLIYNL	9	88.9	Epitope B, Guo et al. 2019, 37-45aa
PA3_Coris-Cartago-CR_2019 liz	22	36	KGRLYRWRSPVIIEK	15	93.3	Gp5-KK, Vashisht et al. (2008), PRRSV2, aa149–163
D7_SanRamon-Alajuela-CR_2019	105	119	KGKLYRWRSPVIIEK	15	86.7	Gp5-KK, Vashisht et al. (2008), PRRSV2, aa149–163
	143	156	TPITKVSAEQWGRP	14	85.7	Gp5-TP, de Lima et al. (2006), PRRSV 2. aa 187–200
	8	17	GTDWLAGKFD	10	90.0	Epitope C Guo et al. 2019, 52-61aa
RA7_Puriscal-SanJose-CR_2019	37	45	SQLQLIYNL	9	88.9	Gp5-SL,Ostrowski et al. (2002), PRRSV2
	149	163	KGKLYRWRSPVIIEK	15	86.7	Gp5-KK, Vashisht et al. (2008), PRRSV2, aa149–163
	52	61	GTEWLAGKFD	10	80.0	Epitope C Guo et al. 2019, 52-61aa

To establish the genetic relationships of isolates from Costa Rica, we constructed phylogenetic trees using seven sequences from field isolates representative of five regions of the country. Sequences from other 10 samples were available but were not included in the study because these sequences were very short or *Cq* values were high (> 30 Ct) suggesting a low presence of viral nucleic acids and therefore not suitable for sequencing. The nucleotide substitution model selected was K2+G and it has the lowest Bayesian Information Criterion (BIC = 11255,8) and described the best substitution pattern [34].

**Fig. 1:**
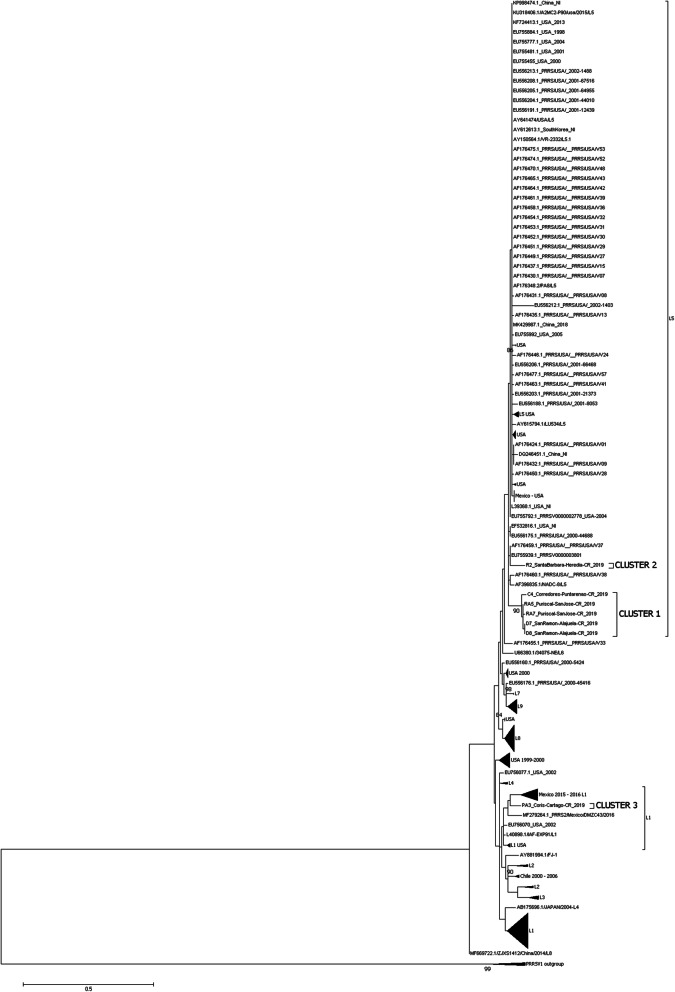
Phylogenetic tree of the ORF5 region of the PRRSV, in production farms in Costa Rica. Evolutionary analysis was inferred by the Maximum Likelihood method based on the Kimura 2-parameter (K2) +gamma model Kimura M. (1980). The tree with the highest log likelihood (-2869,84) is shown. A discrete Gamma distribution was used to model evolutionary rate differences among sites (5 categories (+G, parameter = 0,7491)). The tree is drawn to scale, with branch lengths measured in the number of substitutions per site. The analysis involved 256 nucleotide sequences. All positions containing gaps and missing data were eliminated. Evolutionary analyses were conducted in MEGA7 [34]. Samples from this study (Costa Rica) are denoted by their respective cluster and referred to in the main text. Previously described lineages are identified (L) by brackets

Phylogenetic analysis suggests that Costa Rica sequences are grouped into three different clusters (Fig. 1): cluster 1 (samples from San José, Alajuela, and Puntarenas), cluster 2 (samples from Heredia), and cluster 3 (samples from Cartago). Interestingly, the samples from cluster 1 (San José, Alajuela, and Puntarenas) are related closely (share a common ancestor), despite the geographical distance among these isolates. Sequences from cluster 2 (Heredia) share a common ancestor with strains from the USA. Sequences from clusters 1 and 2 belonged to Lineage 5.

Samples from cluster 3 (Coris, Cartago) displayed a greater genetic distance to the remaining Costa Rican sequences and belonged to Lineage 1. These samples share a common ancestor with strains from Mexico and more distantly of strains from the USA.

## Discussion

For the first time in Costa Rica, the PRRSV circulating variants were characterized based on ORF5 sequence analysis. Among the PRRSV structural proteins, the protein encoded by the ORF5 gene is the most variable and has been used as a marker of genetic variability. Other studies have also built phylogenetic trees based on full-length genomes, non-structural proteins such as the NSP2 gene (one of the most variable regions in the viral genome [35], or more conserved genes such as ORF7 that produces phylogenetic inferences like the derived from the PRRSV full-length genome [36].

Currently, PRRSV genotyping is still performed mostly based on ORF5 and/or ORF7 sequence analysis and ORF-dependent clustering in phylogenetic trees has been observed, possibly associated with recombination [37]. However, there are more ORF5 sequences available on public databases which help to improve the resolution of the phylogenetic relationships.

We anticipate that established PRRSV surveillance and diagnostic systems derived from this work will be adapted to analyze other viral proteins or whole-genome sequencing once information from ORF5 characterization proves to be useful to the swine industry locally.

PRSSV sequences from Costa Rica are grouped exclusively with PRRSV-2. We found three separate clusters of PRRSV-2, suggesting that the virus has three possible origins and/or introductions into the country.

Only a few sequences of PRRSV ORF5 from Central America are described in previous papers and the remaining isolates from the Americas come from Canada, the USA, Mexico, and Chile. Previous research suggests that there are also sequences from Colombia available [38], but we were not able to find them in public databases. In South America, several countries (Argentina, Brazil, Ecuador, Paraguay, and Uruguay) have never reported PRRS disease to the World Organization for Animal Health, whereas in Colombia, Peru, Bolivia, and Venezuela the virus is present [39].

The vast genetic diversity within PRRSV-2 has allowed further classification into 9 lineages based upon ORF5 phylogenetic relationships. These lineages have ~10% nucleotide differences in ORF5 [15, 19]. The most commonly identified PRRSV-2 (>85%) belong to lineages 1, 5, 8, and 9 [19]. Phylogenetic analysis of isolates from Costa Rica are grouped into three different branches within lineages 1 and 5.

A common ancestor for sequences from a Santa Barbara farm is shared with two independent field isolates (EU755939 and AF176459) collected from Illinois, USA in the 90s [40], followed by more phylogenetically distant strains from the USA in the 2000s, all within lineage 5. Interestingly, the movement of PRRSV onto farms does not generally occur via distance-limited processes such as wind or wildlife vectors, but typically occurs via long-distance transport of animals or semen [40]. Although Costa Rican strains were not sequenced until 2019, the first cases of clinical signs associated with PRRSV in Santa Barbara farms were reported in the early 2000s (Table 1), suggesting an introduction of a USA strain in the Costa Rica productive system (unpublished, Santa Barbara farm clinical records).

The sequences from Puntarenas, San José, and Alajuela, are closely related to each other, despite the geographical distance between these provinces. This suggests that these sequences from Alajuela, San Jose, and Puntarenas share a common ancestor with strains isolated in the USA between 1990 and 2000 belonging to lineage 5. In the case of the isolate from Corredores-Puntarenas, farm clinical records included the presence of respiratory and reproductive symptoms in 1996, within approximately one year of an import of breeding stock originating from the USA (unpublished, Corredores- Puntarenas farm clinical records). This clinical history is related to the phylogenetic relationships observed in Fig. 1.

Remarkably, samples from Coris-Cartago are phylogenetically more distant compared to the other samples obtained in Costa Rica. These sequences are grouped closely with several strains from Mexico belonging to lineage 1. The strain DMZC43/2016 is ancestral. Many other sequences classified within lineage 1 and isolates from the USA and Chile were also related to samples from Coris-Cartago, but were phylogenetically more distant. The sample from Cartago is most likely the oldest of the isolates included in this study, because this same farm was positively diagnosed with PRRS in 1993 (León, B. Unpublished data). In addition, in this farm at least five breeders' import events from the USA were recorded from 1988 to 1995. It was assumed that these breeders came from certified pathogen free farms. However, initial detection and important outbreaks of PRRSV in the USA were reported during the late '80s.

The phylogenetic inferences from our study are consistent with previously published trees [19, 39, 41]. The three distinctive strains found in the samples of Costa Rica share common ancestors with USA and Mexican strains. A similar tendency is shown in Mexico [41] and is supported by common trade routes of breeders and semen. It is suggested that genetic similarity between the isolates is not necessarily correlated with the geographical distance. The movement of PRRSV towards the farms is not likely to occur by wind or wildlife vectors, but more commonly through the transport of animals, semen, or fomites [42]. This data reinforces the necessity of conducting PRRSV diagnostic testing before the introduction of animals or semen into a farm and following strict biosecurity measures to prevent transmission of the disease associated with management practices.

Complete or partial amino acid sequences of GP5 from field isolates of Costa Rica were used for epitope research. We described seven different epitope sequences with at least 80% amino acid sequence identity with previously described and experimentally validated immunogenic regions (Table 2) [26, 29, 43]. Importantly, in four out of seven sequences from Costa Rica, we found the conserved peptide (SQLQLIYNL, Table 2) that was previously described as an inducer of IFN-γ. According to Hernandez et al. (2017), in four out of seven pigs vaccinated with the peptide SQLQLIYNL IFN-γ-producing cells were detected and pigs responded to viral challenge [29]. In the future, the use of effective immunogenic regions may help to counteract the meager induction of innate immunity and the slow appearance of virus-specific IFN-γ observed in wild-type infections [25].

Previous studies of the ectodomain of GP5 of PRRSV have detected an immunodominant epitope denominated “A” which is strongly recognized by swine sera early after infection but has no neutralizing activity. In contrast, an epitope denominated “B” is conserved and induces high neutralizing titers. When a pig is infected with PRRSV, epitope A rapidly elicits most of the antibodies directed to GP5 and delays the induction of NAbs against epitope B, thus affecting the ability of the pig immune system to effectively control the infection in an early stage [26]. Interestingly, the epitope search algorithm used in this study was able to map the presence of epitope A, B, and C within the Santa Barbara isolate with an accuracy of ≥ 80%. Although a delay in effective immune responses may be happening in field cases of PRRSV detected in Costa Rica, further experimental evidence is required to validate the decoy effect of Epitope A delaying neutralizing immune responses in field cases.

Future comparison of epitopes from Costa Rica isolates with sequences of commercially available vaccines will help to estimate the in-silico effectiveness of newly introduced vaccines. Findings from our study are relevant as the wild strains described here were circulating before the introduction and use of PRRS vaccines. Future studies will contribute to elucidate how newly introduced vaccines will shape the evolutionary change of circulating field strains.

Currently, PRRSV genotyping is being performed mostly based on ORF5 and/or ORF7 sequence analysis [44]. ORF5 has about 600 nucleotides, shows great genetic diversity, and has a mutation rate of approximately 0.5-1% per year. Although whole-genome sequencing of the PRRSV may give a more complete picture in support of the molecular epidemiology, sequencing ORF5 provides sufficient information to conduct phylogenetic inferences [37, 41].

The PRDC describes the outcome of interactions of multiple respiratory pathogens, environmental factors, type of production system, quality of management, and pig-specific factors (genetics, age, immunological status). One of the limitations in the diagnosis of PRDC is that laboratory assays usually target an individual pathogen and many times there are multiple infectious agents involved in the PRDC [45]. This leads to high expenses for the swine producers as respiratory problems are often not effectively detected and clinical approaches may not be properly installed. In all farms included in this study, we describe the presence of other bacterial and viral pathogens detected during the PRRSV isolation period (Table 1). Interestingly, in the earliest reports of epidemic PRRS cases on all studied farms (1993 to 2015), clinical records showed that PRRS was associated merely with reproductive signs. Later on, during endemic detection of PRRS, cases shifted to be associated with PRDC and major respiratory distress (Table 1). PRRSV is an important agent in the presentation of respiratory problems in pigs. However, the clinical presentation may be complicated by other agents [45]*.* Historical clinical records analysed in this study evidenced the early presence of PRDC in farms from Costa Rica and emphasized the need for better biosecurity and sanitary measures to control respiratory and reproductive clinical disease

Another aspect that may affect disease outcomes is the availability and correct use of effective vaccines. PRRSV vaccines have recently been introduced to Costa Rica. Official records from the Animal Health National Service (SENASA), which is the regulatory authority for drugs and biologicals approved to use for animal health, indicate that vaccines were first approved in 2019. Strains from this study do not show a close phylogenetic relationship with the sequences of vaccine strains included in our phylogenetic tree.

Lastly, research groups that have extensively studied the epidemiology and evolution of PRRSV have provided new insights to reshape veterinary diagnostics by using a full diagnostic platform that incorporates smartphone applications to upload the farm and disease information, and a third-generation sequencing system to identify all viruses and bacteria in the sample within 24 hours [46]. This possibility of rapidly detecting all pathogens present in a sample represents a breakthrough to associate diversity of infectious agents with the severity of the clinical picture.

## Conclusion

While conducting this study, no PRRSV vaccines were approved on the market in Costa Rica. We found PRRSV-2 to be the only species in Costa Rica. For the first time in Costa Rica, a PRRSV-2 phylogenetic analysis was conducted and described based on ORF5 sequence analysis. Field isolates from this study belonged to lineages 1 and 5, according to a well-defined classification system previously described [19]. Our data suggest that PRRSV-2 had at least three different origins or dates of entry into the country. Epitope analysis based on the GP5 sequence of field isolates revealed seven peptides with at least 80% amino acid sequence identity with previously described and experimentally validated immunogenic regions. Future studies are needed to elucidate how newly introduced vaccines, trade of breeders/semen, and management practices (such as biosecurity) define the prevalence and the genetic drift of circulating strains.

## Methods

### Selection of farms and study design

According to the Livestock National Census (2014), there are 14,600 pig holders in Costa Rica, most of which are considered backyard farms. There are ~150 commercial pig farms that produce 80% of the country's total pork production, most of which are located neighboring the metropolitan areas of Costa Rica. The number of sows in Costa Rica is around 39,000 and the number of pigs slaughtered per year is ~780,000. Clinical and management records are available from farms affiliated with the National Chamber of Pig Farmers (*n* = 87).

Using data from a previous seroprevalence study of PRRSV in Costa Rica (manuscript in preparation), we selected PRRSV positive farms to be included in this study. From March to April 2019 (corresponding to the summer season in Costa Rica), ninety samples were collected from 9 farms selected for this investigation to allow the representation of each major Costa Rica region (provinces). Proper animal handling procedures and animal welfare practices were followed to minimize stress, pain, suffering, and distress on sampled pigs. Participating farms had a history of reproductive or respiratory problems in different production stages and have previously been tested for antibodies against PRRSV using ELISA. For the detection and molecular characterization of PRRSV in Costa Rica, samples were screened for the presence of viral RNA by real-time RT-PCR. Eighteen RT-PCR positive serum samples of 8, 10, 12, and replacement ages were selected belonging to representative regions of Costa Rica. Farms were chosen based on seropositivity that was determined in a previous study (manuscript in preparation). In all sampled farms, an epidemiological survey was conducted to determine other laboratory-confirmed respiratory pathogens during the time of sampling for this study. Clinical records were reviewed seeking for first reports and outbreaks of PRRS.

### Screening by RT-PCR

Viral RNA was extracted using the MagMax Pathogen RNA/DNA kit in the MagMAX Express 96 automated equipment (Thermo Fisher Scientific, Pleasanton, CA, USA). The kit VetMAX™ and NA/ EU PRRSV positive controls were used for real-time RT-PCR tests following the manufacturer's instructions [47]. This commercial kit has a sensitivity of 99.5% and a specificity of 99.6%. Samples were processed in a Quant Studio 6 Flex Real-Time PCR thermocycler (Thermo Fisher Scientific, Pleasanton, CA, USA).

### Sanger sequencing

Positive samples determined by the commercial kit were used for RT-PCR and sequencing of amplicons. RT-PCR reactions were carried out using a nested protocol with the internal and external primers (PRRS OUT and PRRS IN) described in Table 1 as described before [48]. Locations of primers are based on PRRSV Yamagata10-7 GenBank: AB811788.1.

Briefly, superscript III One-Step RT-PCR Platinum Taq HiFi reagents (Thermo Fisher Scientific, Pleasanton, CA, USA) were used following the manufacturer's instructions and run on a Veriti 96 well Thermal Cycler equipment (Thermo Fisher Scientific, Pleasanton, CA, USA). The amplified DNA products were run on a 2% agarose, bands of the expected size were excised and purified using the QIAquick Gel Extraction kit (Qiagen, Valencia, CA, USA), according to the manufacturer specifications.

Purified DNA was quantified by using a Nanodrop ® (Thermo Fisher Scientific, Pleasanton, CA, USA). For each sample, purified forward and reverse amplicons were used for sequencing using BigDye Terminator v3.1 Cycle Sequencing kit (Thermo Fisher Scientific, Pleasanton, CA, USA), with a final volume of 20 μL and run on a Veriti 96 well Thermal Cycler equipment (Thermo Fisher Scientific, Pleasanton, CA, USA). The sequencing cycling conditions were the following: 2 min. 96 ° C, 30 cycles (10 sec. 96 °C; 5 sec. 50 °C; 4 min. 60 °C). Purification of the sequencing reaction was carried out with the BigDye XTerminator Purification Kit (Thermo Fisher Scientific, Pleasanton, CA, USA) and the final sequencing of the products was performed on an ABI3130 sequencer (Thermo Fisher Scientific, Pleasanton, CA, USA).

Chromatographic curves were analyzed using Sequencing Analysis 5.4 software (Thermo Fisher Scientific, Pleasanton, CA, USA) and subsequently assembled using SeqScape 2.6 software (Thermo Fisher Scientific, Pleasanton, CA, USA).

### Phylogenetic analysis

The sequences obtained in this study (*n* = 7) were compared against other previously published sequences corresponding to PRRSV ORF 5 downloaded from public sequence repositories (GenBank / DDBJ / EMBL, Table 3). All accession numbers of samples included in this study (BankIt2396380 D8_SanRamon-Alajuela-CR_2019 MW186701, BankIt2396380 RA5_Puriscal-SanJose-CR_2019 MW186702, BankIt2396380 D7_SanRamon-Alajuela-CR_2019 MW186703, BankIt2396380 C4_Corredores-Puntarenas-CR_2019 MW186704, BankIt2396380 RA7_Puriscal-SanJose-CR_2019 MW186705, BankIt2396380 R2_SantaBarbara-Heredia-CR_2019 MW186706, BankIt2396380 PA3_Coris-Cartago-CR_2019 MW186707) as well as historical and reference strains [49] are depicted in the phylogenetic tree and aligned using the Clustal program [47].

**Table 3:** Primers used for sequencing of ORF5

Name	Sequency	AB811788	Amplicon size
PRRS OUT 1 F	5’- GTACGGCGATAGGGACACC-3´	13416	
PRRS OUT 2 R	5’- CCAGAATGTACTTGCGGCC-3	14672	1256 pb
PRRS P420 F	5´- CCATTCTGTTGGCAATTTGA -3´	13731	
PRRS P620 R	5´- GGCATATATCATCACTGGCG-3´	14440	716 pb

The aligned sequences were manually curated using the Bioedit program [50]. Identical sequences according to the pair distance algorithm of the MEGA 7 program [34] removed from the analysis and the remaining 252 sequences were used for phylogenetic analysis. The phylogenetic tree was constructed, using the Maximum likelihood distance method, of the MEGA 7 program [34], with a bootstrap of 1000 repetitions, and PRRSV-1 sequences Lelystad (M96262.2), Lena (JF802085.1), and Belgium 2013 (KT159249) were used as external groups. The "Find the best model" tool incorporated in MEGA 7 was used to determine which substitution model best fit the sequences selected for each of the strains analyzed. The Kimura 2-parameter + G (K2+G) replacement model was used, under the Bayesian Information Criterion (BIC). Initial tree(s) for the heuristic search were obtained automatically by applying Neighbor-Join and BioNJ algorithms to a matrix of pairwise distances estimated using the Maximum Composite Likelihood (MCL) approach and then selecting the topology with a superior log-likelihood value. The tree with the highest log-likelihood is shown and bootstrap values above 70% are displayed. All positions containing gaps and missing data were eliminated.

### Epitope analysis

To identify the presence of potential conserved epitopes across circulating strains in Costa Rica, we utilized a database of experimentally validated peptides previously published [26, 29, 43] and compared these motifs with the amino acid sequence of isolates from Costa Rica.

Sequences from Costa Rican isolates were trimmed, compared, and aligned using CLC Genomics Workbench 10.1.1. A predicted ORF5 amino acid sequence for each experimental sequence was generated. Experimental ORF5 amino acid sequence was queried compared to previously published and experimentally validated epitopes [29, 51] (Supplementary File 1) using the motif search tool of CLC Genomics Workbench 10.1.1. Settings were configured to allow matching with a minimum of 80 % sequence identity for epitope prediction.

## Supplementary Information

**Additional file 1.**

## Data Availability

The data sets analyzed during the current study are available from the corresponding author upon reasonable request. New sequences derived from this study were deposited at the NCBI repository and the corresponding accession numbers were indicated in the manuscript.

## Abbreviations

APP: *Actinobacillus pleuropneumoniae*
Cq: quantification cycle
Ct: threshold cycle
DNA: Deoxyribonucleic acid
EU: European Union
GP5: Glycoprotein 5
ICTV: International Committee on Taxonomy of Viruses
IFN-γ: gamma interferon
MLV: Modified Live Virus
NA: North American
NAbs: Neutralizing Antibodies
NSP: Non-structural protein
ORF: Open Reading Frame
(RT)PCR: (Real Time) Polymerase Chain Reaction
PRRS: Porcine Reproductive and Respiratory Syndrome
PRDC: Porcine Respiratory Disease Complex
RNA: Ribonucleic acid
